# An evaluation of an open access iPSC training course: “How to model interstitial lung disease using patient-derived iPSCs”

**DOI:** 10.1186/s13287-023-03598-9

**Published:** 2023-12-20

**Authors:** Anja Schweikert, Sarah Kenny, Irene Oglesby, Arlene Glasgow, Chiara de Santi, Ingrid Gensch, Nico Lachmann, Tifenn Desroziers, Camille Fletcher, Deborah Snijders, Nadia Nathan, Killian Hurley, COST Open-ILD Group Management Committee, Deborah Snijders, Nicolaus Schwerk, Nico Lachmann, Matthias Griese, Daniel O’Toole, Raphael Borie

**Affiliations:** 1 Department of Medicine, Royal College of Surgeons in Ireland, Education and Research Centre, Beaumont Hospital, Dublin 9, Ireland; 2 Tissue Engineering Research Group, Royal College of Surgeons in Ireland, Dublin 2, Ireland; 3 School of Medicine, Trinity Biomedical Sciences Institute, Trinity College Dublin, Dublin 2, Ireland; 4 Department of Clinical Microbiology, Royal College of Surgeons in Ireland, Dublin 9, Ireland; 5 School of Pharmacy and Biomolecular Sciences, Royal College of Surgeons in Ireland, Dublin 2, Ireland; 6 Department for Pediatric Pneumology, Allergology and Neonatology, Hannover Medical School, Hannover, Germany; 7 Cluster of Excellence – Resolving Infection Susceptibility (RESIST, EXC 2155), Hannover Medical School, Hannover, Germany; 8 Biomedical Research in Endstage and Obstructive Lung Disease Hannover (BREATH), German Center for Lung Research, Hannover, Germany; 9 Regenerative Biology to Reconstructive Therapy (REBIRTH) Center for Translational and Regenerative Medicine, Hannover Medical School, Hannover, Germany; 10 Laboratory of Childhood Genetic Disorders Inserm UMR_S933, Armand Trousseau Hospital, Sorbonne University, Paris, France; 11 Department of Woman and Child Health (SDB), Primary Ciliary Dyskinesia Centre, University of Padova, Padua, Italy; 12 Pediatric Pulmonology Department and Reference Centre for Rare Lung Diseases RespiRare, Armand Trousseau Hospital, APHP Sorbonne University, Paris, France

**Keywords:** iPSCs, Interstitial lung disease, Disease modelling, European Cooperation in Science and Technology, Training course, COST Action IG16125

## Abstract

**Background:**

Interstitial lung diseases (ILD) are a group of rare lung diseases with severe outcomes. The COST Innovator Grant aims to establish a first-of-a-kind open-access Biorepository of patient-derived induced pluripotent stem cells (iPSC) and to train researchers in the skills required to generate a robust preclinical model of ILD using these cells. This study aims to describe and evaluate the effectiveness of a training course designed to train researchers in iPSC techniques to model ILD.

**Methods:**

74 researchers, physicians and stakeholders attended the training course in Dublin in May 2022 with 31 trainees receiving teaching in practical iPSC culturing skills. The training course learners were divided into the Hands-on (16 trainees) and Observer groups (15 trainees), with the Observers attending a supervised live-streamed experience of the laboratories skills directly delivered to the Hands-on group. All participants were asked to participate in an evaluation to analyse their satisfaction and knowledge gained during the Training Course, with means compared using *t*-tests.

**Results:**

The gender balance in both groups was predominantly females (77.4%). The Hands-on group consisted mainly of researchers (75%), whereas all participants of the Observer group described themselves as clinicians. All participants in the Hands-on group were at least very satisfied with the training course compared to 70% of the participants in the Observer group. The knowledge assessment showed that the Hands-on group retained significantly more knowledge of iPSC characteristics and culturing techniques compared to the Observers (* < 0.05; *p* = 0.0457). A comprehensive learning video detailing iPSC culturing techniques was produced and is included with this manuscript.

**Conclusions:**

The majority of participants were highly or very satisfied with the training course and retained significant knowledge about iPSC characteristics and culturing techniques after attending the training course. Overall, our findings demonstrate the feasibility of running hybrid Hands-on and Observer teaching events and underscore the importance of this type of training programme to appeal to a broad spectrum of interested clinicians and researchers particularly in rare disease. The long-term implications of this type of training event requires further study to determine its efficacy and impact on adoption of iPSC disease modelling techniques in participants’ laboratories.

**Supplementary Information:**

The online version contains supplementary material available at 10.1186/s13287-023-03598-9.

## Introduction

Interstitial lung disease (ILD) in adults are a group of devastating fatal lung diseases leading to scarring of the lung and death often within 3 years of diagnosis [1]. In children chILD are severe diseases with variable prognosis and eventual fibrosing evolution [2]. New antifibrotic medications offer hope of slowing disease progression in adults [3, 4], however, side effects and hepatic toxicity caused by the approved drugs often lead to discontinuation of medication by the patient [4]. Therefore, no proven effective cures are currently available for children, with lung transplant being the only effective option in end-stages lung fibrosis. [4] Genetic factors may cause or contribute significantly to the risk of developing ILDs and those patients with inherited forms of ILD may have a worse prognosis than sporadic-ILD, respond poorly to current treatments and some individuals may have serious adverse reactions to immunosuppression after transplantation [5, 6].

Across Europe diverse mutations including telomere-related genes (TRG), predominantly in adults, and more rarely surfactant related genes (SRG), mainly in children can lead to abnormal cell ageing and surfactant protein processing in lung alveolar epithelial cells, respectively [2, 7, 8]. The rarity of individual mutations contributes to a lack of basic mechanistic studies and randomised control trial data on effectiveness of treatments. Consequently, management strategies derived from other diseases are based on physicians' experience and remain controversial.

The current-state-of-the-art preclinical models fail to accurately recapitulate the diverse genetic causes of ILD. Animal models, despite contributing to the development of new therapeutics cannot recapitulate the various disease presentations or progressive pathology, which characterises the disease in humans. For example, exposure of mice to Bleomycin, the main preclinical model used to date cannot predict the clinical efficacy of candidate human therapeutics [9]. Human ex vivo models like precision-cut lung slices are often only explanted from patients with end-stage fibrotic disease [10] limiting the study possibility of early disease. Together these limitations of existing preclinical models limit researchers’ access to tissue that faithfully recapitulates normal and abnormal human lung biology creating a major hurdle to identifying and personalising treatments for patients. Induced pluripotent stem cells (iPSCs) are poised to overcome these hurdles as they contain the unique genetic background of each individual, and have the capacity for differentiation to relevant somatic cells. We have previously shown that we can employ patient-derived gene-edited iPSCs to generate alveolar epithelial cells and macrophages and successfully model ILD and other chronic lung diseases in vitro [11–14]. However, widespread adoption of these models in laboratories across the field has been slow due to several technical challenges. Therefore, we trained researchers in iPSC culturing techniques as part of a training course and prepared video protocols and a repository of research resources (Additional files 1 and 2) that will help researchers to set foot in the iPSC field.

The training course was funded by the COST Innovator Grant “Open-ILD: An Open Access Repository of Pluripotent Stem Cells from Children and Adults with Interstitial lung disease” followed the COST Action European Network for Translational Research in Children’s and Adult ILD (ENTeR chILD). ENTeR chILD, a network of multidisciplinary clinicians (paediatric and adult), scientists, and patients and their families, initiated a pan-European research network with the ultimate goals of delivering accurate and early diagnosis with structured, personalised, management and therapies. COST (European Cooperation in Science and Technology) is an EU-funded programme that was created to enable researchers to establish interdisciplinary research networks in Europe and other affiliated countries [15].

The aim of Open-ILD is to leverage this existing transdisciplinary network to establish a first-of-a-kind, Europe-wide open-access repository of patient-derived iPSC linked with highly phenotyped patient data to fill the unmet need for better preclinical models of ILD, thereby allowing for the identification of novel therapies and modes of delivery. The establishment of this resource will foster a growing body of research across Europe to develop a robust preclinical model of ILD that can (a) explore pathogenesis (b) identify new patient-specific druggable targets and (c) test novel compounds and inhaled modes of delivery for patients in vitro before clinical studies, leading to shortening of drug development pipelines and reduction of toxic side effects.

One aim of the Open-ILD is to generate protocols and standard operating procedures for working with iPSC and differentiating iPSC to lung organoids and macrophages. Additionally, during a training course, researchers were trained in the skills required to work with these cells to generate a robust preclinical model of ILD by expert members of the working group. This paper will detail the proceedings of the training course which occurred in Dublin in May 2022, measure and analyse the attendees’ knowledge of the skills and information taught and summarise the outcomes of the training course. Importantly, this paper offers a manual summarising the protocol (Additional file 2) and video protocols (Additional file 1) to help researchers in starting iPSC work.

## Methods

### Description of the training course

The training course was composed of Theoretical Sessions giving background information about the learning material and Practical Sessions. It was held over two days at the RCSI Education & Research Centre, Smurfit Building, Beaumont Hospital. The Practical Sessions were delivered by a novel Hands-on/Observer Hybrid Model. Due to space constraints and the high number of applicants, attendees were divided into two cohorts, namely “Hands-on” (16 trainees) and “Observers” (15 trainees). Allocation to these groups was dependent on the trainees’ previous laboratory-based experience (particularly in cell culture) and/or their area of research interest (e.g. pre-clinical modelling of lung disease). A camera was set up in one of the Class II Biosafety hoods where the trainers and Hands-on group were working, enabling a live video stream of the practical session to the Observer group in a lecture hall elsewhere in the building. The camera was positioned to record the cell culture techniques (now edited with added narration and available in Additional file 1), and a sound link allowed constant communication between the trainer in the cell culture room and the trainer/expert leading the Observer session. Members of the Observer group could therefore view and hear the practical sessions, as well as ask questions to the trainer.

Each trainee was given a training booklet covering the learning objectives and all the protocols of the practical sessions that can be found in the supplements of this paper (Additional file 2). The Learning Objectives of the Practical Sessions are shown in Table 1. The first day of the training course included lectures introducing participants to the basic principles and techniques of iPSC culture and directed differentiation to alveolar cells and to macrophages. These were interspersed by three practical sessions where trainers demonstrated (i) passaging of iPSC, (ii) thawing of iPSC, and (iii) freezing of iPSC. In each of these sessions, members of the Hands-on group were divided into three smaller groups of 5–6 people each and went to one of three cell culture laboratory rooms with an assigned trainer. The trainer demonstrated the protocol, explaining each step and including details/tips that are often difficult to relay in a published protocol. These tips and details are summarised in our video protocols (Additional file 1). The Hands-on trainees then had a chance to practice the technique themselves, while the trainer provided guidance and answered questions from the group. In a fourth practical session, the first step of directed differentiation towards definitive endoderm was demonstrated by the trainers.

**Table 1:** Learning objectives of the practical sessions

Session	Learning objective
A	To understand the principles and techniques for culturing iPSCs (based on the introductory lecture)
	To understand the practical aspects of (i) how to coat plates with 2D Matrigel™ for iPSC culture, and (ii) how to passage iPSC on Matrigel using Gentle Cell Dissociation Reagent
B	To understand the theoretical and practical aspects of thawing frozen stocks of iPSCs onto 2D Matrigel
C	To understand the theoretical and practical aspects of making frozen stocks of iPSCs for long-term storage
D	To understand the principles and mechanisms behind generating endoderm and subsequently lung progenitors and distal lung organoids from iPSCs using a directed differentiation protocol
	To understand the principles and mechanisms involved in generating macrophages from iPSCs
	To understand the practical aspects of preparing/seeding iPSCs for differentiation towards endoderm
E	To recognise the expected microscopic appearance and characteristics of (i) iPSCs passaged in Session A; (ii) iPSCs thawed in Session B; and (iii) iPSCs seeded for endoderm differentiation in Session D
F	To examine by microscope: (i) iPSC-derived macrophages, and (ii) iPSC-derived lung organoids

On the second day, both the Hands-on and Observer participants had the opportunity to examine by microscope the thawed and passaged iPSCs from the previous day. Trainers advised on the quantity and quality of the colonies, and checked for spontaneous differentiation. Trainers facilitated an interactive discussion, giving advice and feedback on what was observed. Previously prepared iPSC-derived macrophages and lung organoids were also available so that the trainees could see what the end product of differentiation would look like.

### Implementation of a trainee survey and evaluation

After completion of the training course, all participants were asked to fill in an anonymous evaluation questionnaire (Additional file 3). All 16 participants of the Hands-on group participated in the survey as did 10 of the 15 attendees of the Observer group. The evaluation form was divided into four parts: (A) four questions about the attendee’s demographics (career position, age, pre-existing knowledge); (B) a survey of satisfaction with the training sessions generally and in terms of knowledge gained consisting of 14 questions with response scores from 1 to 10 or 1 to 6; (C) two questions on the trainee’s self-estimation of knowledge improvement (scored from 0 to 10); and (D) ten multiple choice questions assessing the trainee’s knowledge of the characteristics of iPSCs and their culturing techniques. We gathered and analysed the data within four weeks of the training course finishing. One question in part D (knowledge assessing question) was excluded from analysis due to a high similarity between proposed choices leading to multiple correct answers. For analysis purposes, the scores were divided into highly satisfied (9–10 out of 10; 6 out of 6), very satisfied (7–8 out of 10; 5 out of 6), moderately satisfied (4–6 out of 10; 3–4 out of 6) and not satisfied (1–3 out of 10; 1–2 out of 6). Results of Hands-on participants and Observers were analysed separately, using a security protected Microsoft Excel database and graphs were drawn using GraphPad Prism. Means were compared using *t*-tests.

## Results

### Characteristics of training course attendees

**Fig. 1:**
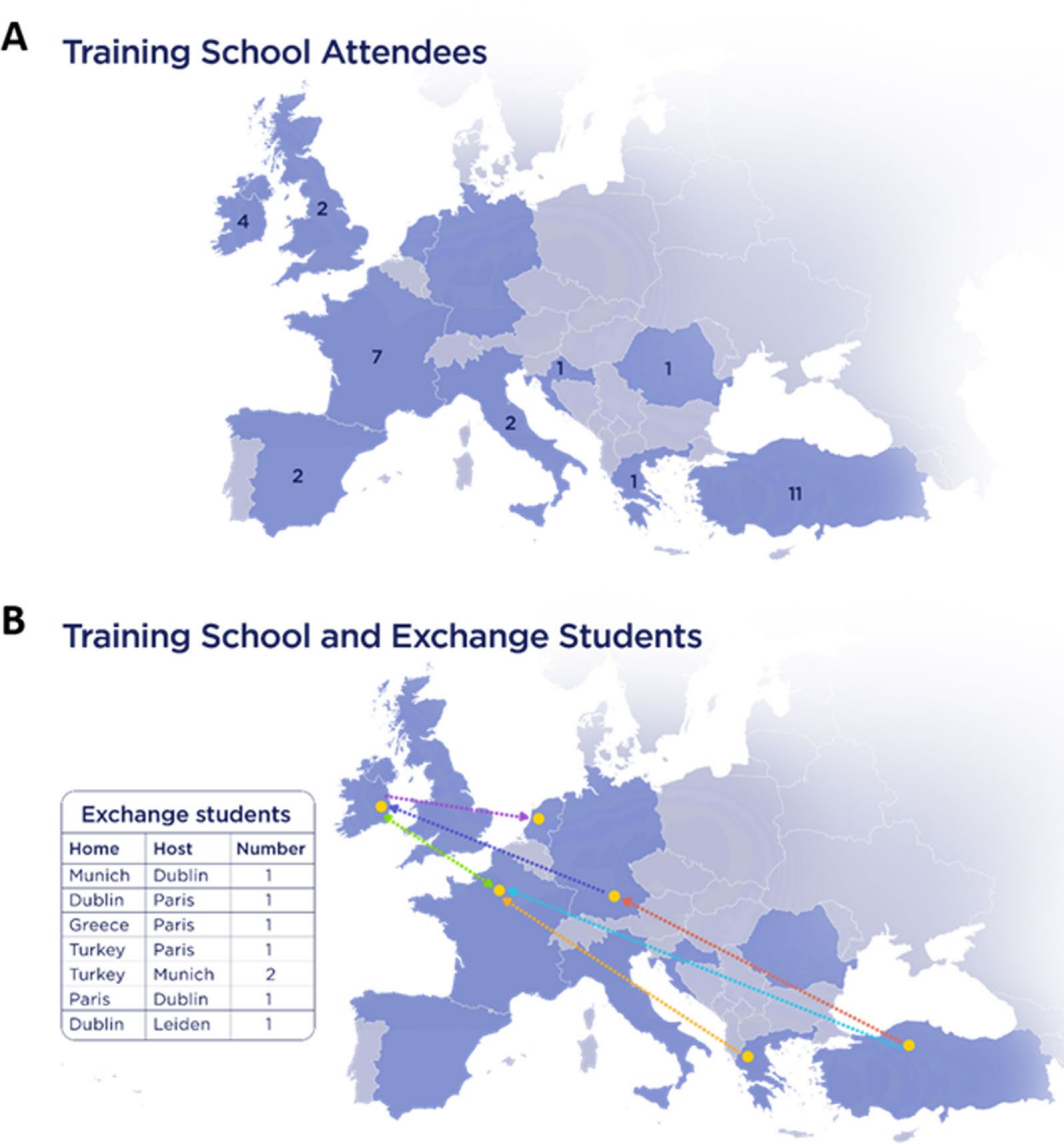
**Origin of training course attendees**. **A** A map identifying countries across the European Research Area from which attendee’s host institutions were located (*n* = 31). **B** A map showing the countries where researchers travelled to for Short-Term Scientific Missions (*n* = 8)

**Fig. 2:**
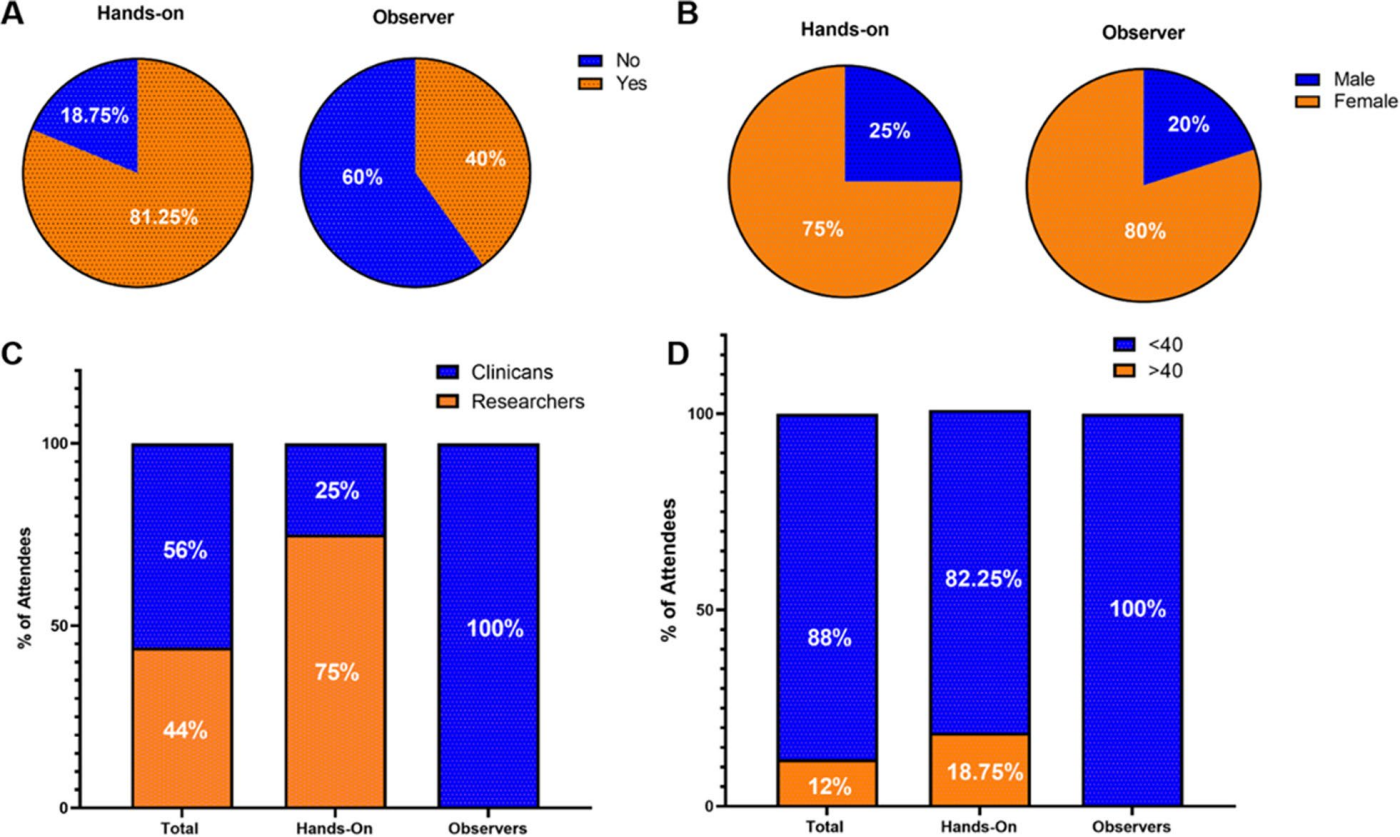
**Analysis of training course attendees**. **A** Previous laboratory-based experience in cell culture in the Hands-on (*n* = 16) and the Observer group (*n* = 10) based on survey results. **B** Gender distribution of all attending trainees in the Hands-on (*n* = 16) and Observer group (*n* = 15) based on inscription data. **C** Professional distribution of the trainees in the Hands-on and Observer group in %. (*n* = 26; 12 Researcher, 14 Clinicians) based on survey results. **D** Age distribution (< 40 years of > 40 years) of trainees in the Hands-on and Observer group in % (*n* = 26; 23 < 40, 3 > 40) based on survey results

We analysed the characteristics of the training course attendees based on the information gained upon registration and the survey results. A total of 33 out of 44 candidates, representing 10 different countries (Fig. 1), were accepted to attend the training course. 31 candidates attended as two candidates could not travel due to COVID-19 and visa restrictions. The trainees were divided into the Hands-on training group (*n* = 16) and the Observer group (*n* = 15) with 81.25% of participants of the Hands-on cohort and 40% of the Observer group reporting previous laboratory-based experience in cell culture (Fig. 2A). Women represented 77.4% of the trainees (Fig. 2B). All trainees from the Hands-on group (100%) and 10 trainees of the Observer group (66.7%) participated in the survey. The Hands-on group was composed of 12 researchers (75%) and four clinicians (25%), whereas all 10 participants of the survey (100%) in the Observer group described their position as Clinician (Fig. 2C). In total, all but three participants in the Hands-on group (81.25%) were aged < 40 years and all trainees in the Observer group who completed the survey were < 40 years (100%) (Fig. 2D).

### Hands-on group satisfaction with the training course and evaluation of their knowledge

**Fig. 3:**
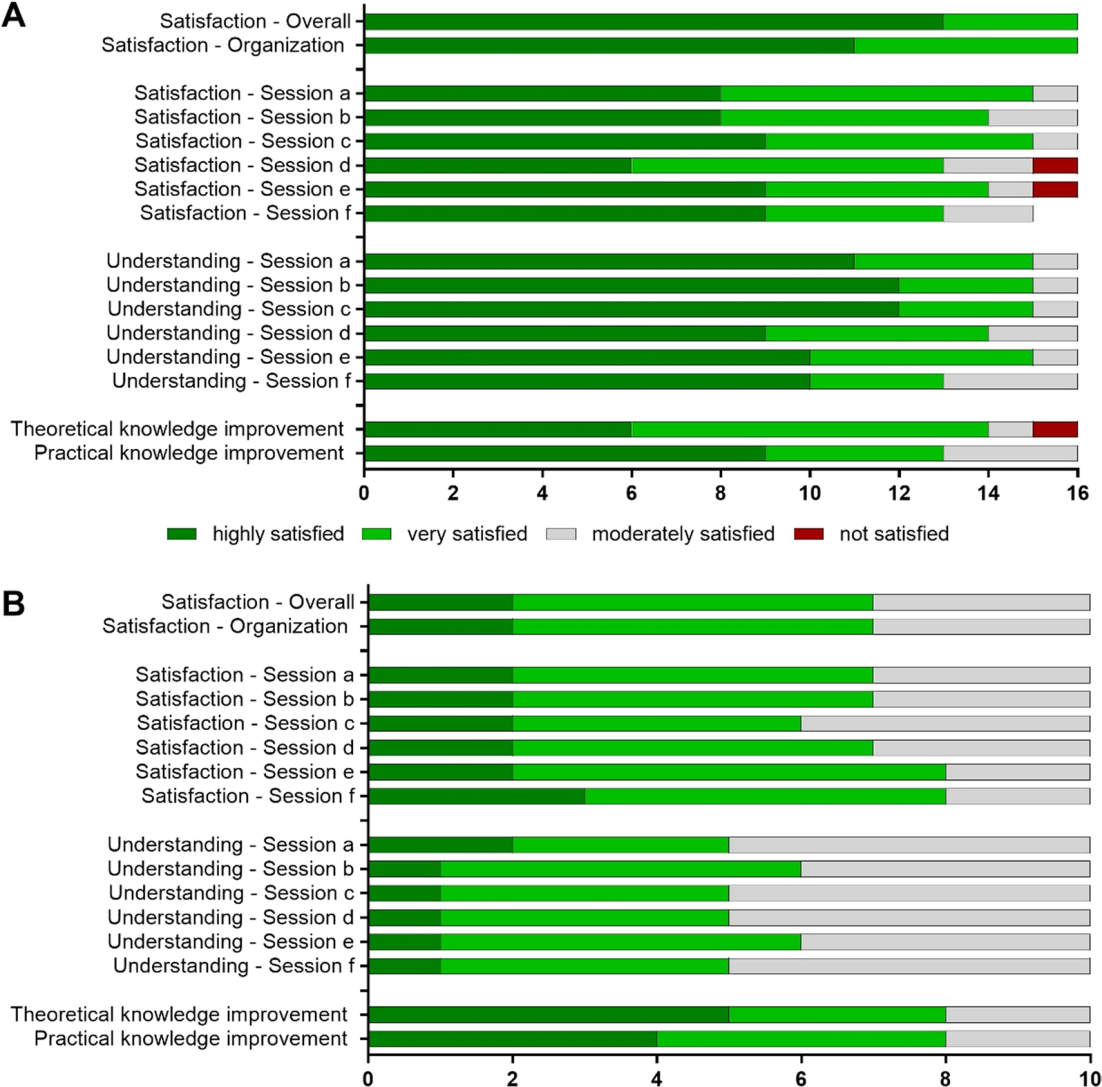
**Trainee satisfaction with the training course.** Survey of trainee satisfaction with the training course for **A** Hands-on trainees (*n* = 16) and **B** Observers (*n* = 10). (Session a) Basics of iPSC Culture and Maintenance; (Session b) Thawing iPSCs; (Session c) Freezing iPSCs; (Session d): Differentiation of iPSCs; (Session e) Evaluation of iPSC Culture and Differentiation, (Session f) Evaluation of iPSCs, Macrophages and Lung Organoids

We next assessed the attendee’s satisfaction with the practical Hands-on session using an anonymous survey applied directly after the training course. The satisfaction could be rated from 1 to 10 and the responses were grouped into highly satisfied [9, 10], very satisfied [7, 8], moderately satisfied [4–6] and not satisfied [1–3]. All the participants in the Hands-on group were at least very satisfied or better (*n* = 16, 100%) with the in-lab sessions in general as well as their organisation (*n* = 16, 100%) (Fig. 3A). Additionally, the participants self-evaluated their degree of satisfaction and understanding of the individual sessions (A: Basics of iPSC Culture and Maintenance, B: Thawing iPSCs, C: Freezing iPSCs, D: Differentiation of iPSCs, E: Evaluation of iPSC Cultures and Differentiation and F: Evaluation of iPSCs, Macrophages and Organoids). Here, the answers ranged from 1 to 6, with 6 being highly satisfied, 5 very satisfied, 3–4 moderately satisfied and 1–2 not satisfied. In average the participants were very satisfied (score 5.3 out of 6) with all sessions. However, Session D only scored a mean of 5 out of 6 (very satisfied). Sessions A, B and C scored the highest with a mean of 5.4 (very satisfied).

The evaluation of the trainees' understanding of the individual training sessions showed that the mean score for all sessions combined was 5.6 (highly satisfied). Session D and F scored the lowest with a mean of 5.4 (very satisfied) and Session B and C scored the highest with a mean of 5.7 (highly satisfied).

Additionally, trainees self-evaluated their overall improvement in practical and theoretical knowledge of iPSCs culturing techniques after the training course on a scale from 1 to 10. Overall, the majority of the participants (*n* = 13, 81.25%) were very satisfied or better with their practical knowledge improvement. When self-evaluating their theoretical knowledge improvement, 14 participants (87.5%) were at least very satisfied (Fig. 3A).

**Fig. 4:**
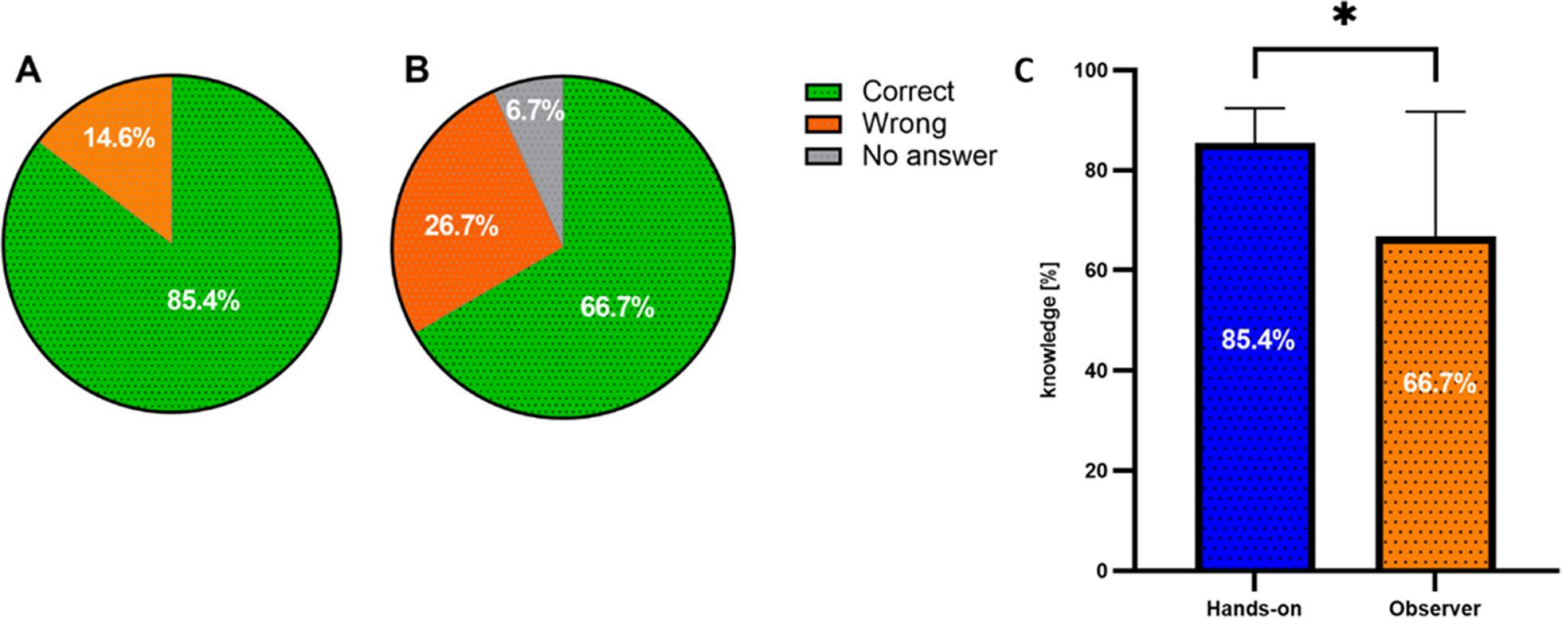
**Knowledge of trainees after the training course.** Knowledge survey after the training course for **A** Hands-on group of trainees (*n* = 16) and **B** Observer group of trainees (*n* = 10). **C** *t*-Test of knowledge about iPSC culture of Hands-on cohort (*n* = 16) vs. Observer group (*n* = 10) as result of 9 multiple choice knowledge questions in the survey (* < 0.05; *p* = 0.0457)

Finally, the trainees' knowledge of the characteristics of iPSC and techniques used to culture cells post training course was assessed using 9 multiple choice questions (MCQs) set by a team of experts from Open-ILD. Participants in the Hands-on group answered 85.4% of the questions correctly with no questions left unanswered (Fig. 4A). The Hands-on group answered significantly better than the Observer group when counting unanswered questions as incorrect (85.4% vs 66.7%, *t*-test *p* = 0.045, Fig. 4C).

### Observer group satisfaction with the training course and evaluation of their knowledge

Using the same survey, we assessed the satisfaction of the participants within the Observer group. To compare the efficiency and satisfaction of both groups, their results were analysed separately. The majority of participants in the Observer group reported that they were at least very satisfied (*n* = 7, 70%) with the Observational sessions in general (Fig. 3B). The same results were seen for the organisation of the Observational sessions (*n* = 7, 70% very satisfied or higher). When scoring the satisfaction with the individual sessions, the average score was 4.7 (very satisfied). Session C (Freezing iPSCs) scored lowest with 4.6 (very satisfied), while Session F (Evaluation of iPSC, Macrophages and Lung Organoids) reached the highest score with 4.9 (very satisfied).

The same trend was seen in the self-evaluation of their understanding of the individual sessions. The mean score for all sessions was 4.5 (very satisfied). The highest mean score of 4.6 (very satisfied) was seen for session E (Evaluation of iPSC Culture and Differentiation) and Session C (Freezing of iPSCs) again reached the lowest score of 4.4 (moderately satisfied).

In contrast, when trainees self-evaluated their personal theoretical and practical knowledge improvement, the majority of the participants stated they were at least very satisfied (*n* = 8, 80%). Finally, when evaluating the Observer group’s knowledge of iPSC characteristic and culturing techniques using MCQ’s, results show that 66.7% gave correct answers and 6.7% did not answer specific questions. Compared to the Hands-on group, the Observer group had a significantly lower score (Fig. 4C, *t*-test *p* = 0.0457).

## Discussion

We established the first-of-its-kind multimodal hybrid training course to teach researchers to work with iPSCs to generate preclinical models of ILD. This training course funded by COST consisted of 'Hands-on' (in the laboratory) and 'Observer' groups in an adjacent tutorial room linked by live steaming video and audio. During the practical laboratory sessions iPSC culturing techniques such as thawing, passing and freezing were demonstrated and focused lectures were delivered to both groups. This multimedia report, together with accompanying video protocols (Additional file 1) aims to describe the training course, the profile of the researchers attending, their previous experience, their satisfaction with the teaching andan evaluation of knowledge gained at the training course. This report also provides a resource of video protocols (Additional file 1) and a repository of research protocols (Additional file 2) to researchers aiming to work with iPSC to generate preclinical models of ILD. The results show that the approach employed to teach iPSC-culturing skills and understanding is feasible, effective and delivers a high degree of satisfaction for participants. This study also demonstrates that trainees retained a high degree of understanding of iPSC culturing techniques, rationale for their use and the application of iPSC-derived macrophages and lung organoids as preclinical models of ILD.

We found that all participants in the Hands-on group (*n* = 16, 100%) and the majority in the Observer group (*n* = 7, 70%) were at least very satisfied with the training course overall (Fig. 3). Previously, a series of training courses held as part of the BM1303-DXDNet COST Action, demonstrated similar high trainee satisfaction [16]. When examining scoring of the individual sessions a mean of 85.63% (*n* = 13.7) of the Hands-on cohort reported being at least very satisfied compared to 72% (*n* = 7.2) of the Observer cohort. The understanding of the individual sessions was rated at least very satisfying by a mean of 90.63% (*n* = 14.5) in the Hands-on group but only 53.3% (*n* = 5.33) of the Observer group. This difference in satisfaction with understanding may be due to the higher levels of previous laboratory experience reported in the Hands-on group (Fig. 2A) as well as the difference in reported profession (Researcher or Clinician, Fig. 2C). These differences between Hands-on and Observer Groups were also reflected in the result of the analysis of the knowledge questions (Fig. 4) where significantly more attendees in the Hands-on group answered the questions correctly compared to the Observer group (85.4 vs 66.7%, *p* = 0.0457) (Fig. 4C). However, these results may reflect baseline differences in knowledge in the Hands-on versus the Observer Groups. Overall, these findings support the benefits of a training course model, while also suggesting that in future training courses, special attention should be given to the pre-existing knowledge of individual participants. Additional background information could be delivered to those that have less knowledge before the start of the training course.

The Hands-on/Observer hybrid model employed in this training course is commonly used in surgical education and found particular utility during the COVID-19 pandemic where restrictions severely limited in-person training [17]. In the surgical model surgeries and procedures are live-streamed and real-time communication between the surgeon and students is possible. Studies have shown the potential of this model to improve surgical education [18, 19]. However, the use of this hybrid model in a laboratory-based setting has not been reported to date. Our study evaluated the feasibility, trainee satisfaction and knowledge retention offered by this method in the laboratory setting. Additionally, in line with the goals of WG2, this hybrid model allowed for the development of video protocols (Additional file 1) which will be of great use to researchers beginning to work with iPSCs, thus highlighting the flexibility and benefits of this novel hybrid model.

Over three quarter of the participants in the COST Open ILD training course were female. Additionally, 88% of the participants were younger than 40 years. Of note, these numbers do not include the trainees that were not taking part in the survey. A study by Plank-Bazinet and colleagues shows that women are well represented in doctoral degree programmes but account for less than half of the academic medicine faculty positions [20]. In addition, a survey of the Association of American Medical Colleges entitled ‘The State of Women in Academic Medicine’ found that although more women than men are enrolled in Biological and Medical Sciences Doctorate Programs, the number of male post doctorates in this field is higher compared to female [21]. The same trend towards male postdoctoral scientists in Biological and Biomedical sciences was found by the National Center for Science and Engineering Statistics [22]. Consistent with the literature we found that while 75% of the participants in the practical part of the training course were female only 40% of the speakers invited to the lecture series section of the training course were female (Additional file 4: Fig. S1).

As a result of the networking opportunities obtained at the training course eight short term scientific missions (STSM) were agreed and arranged. These STSMs funded eight early-stage researchers to travel to laboratories or clinical services across the European Research Area (ERA) to carry out research in topics related to the aims of the CIG. These STSMs included projects related to further training in iPSC and other cellular models of ILD and assessing diagnostics and clinical services for patients with chILD. As of the end of the one year term of CIG and 6 months after the training course, newly established collaborations have supported successful grant applications to the 2022 European Research Council, abstracts to the European Respiratory Society (ERS) Congress in Barcelona 2022 and Milan 2023 and numerous opportunities for future grant applications and publications. Furthermore, new connections have been established with other COST Actions including CA20140—CorEuStem: The European Network for Stem Cell Core Facilities, the European ILD Registry and Biobank (eurILDreg) and the ERS Children’s Interstitial lung disease Clinical Research Collaboration (ChILDEU CRC). This training course in conjunction with the COST Action CA16125 has led to the establishment of three new European wide cohorts of patients with chILD including those focused on (a) Neuroendocrine Cell Hyperplasia of Infancy (NEHI), (b) ABCA3 mutation related chILD and (c) diffuse alveolar haemorrhage (DAH).

### Recommendations for those running a practical laboratory skill-based training course

- Keep the trainer to trainee ratio small to ensure optimal supervision.
- A hybrid teaching model of a Hands-on group and Observer group is feasible and gives more participants the opportunity to learn about the cell culture techniques while allowing more tailored information to be delivered to those with and without prior cell culturing experience.
- Dividing the practical laboratory sessions into short interactive sessions allows trainees enough time for questions and discussions improving knowledge and skills acquisition.
- Providing a Laboratory Handbook containing protocols before the training course improves satisfaction and knowledge acquisition (Additional file 2).
- Delivering important background information in introductory lectures before the corresponding practical session supports trainees with less pre-existing knowledge of the subject.

### Limitations

Several limitations to this study are important to highlight. While this study aims to investigate the immediate satisfaction and knowledge retention of the trainees after the training course it is possible that differences in knowledge improvement between the Hands-on and Observer groups is due to pre-existing knowledge in the more experienced, researcher predominant Hands-on group. The Hands-on group described themselves as having more general cell culture experience and self-identified as researchers rather than clinicians. However, only two participants in the Hands-on group report having specific iPSC culture experience. To further investigate the important medium and long-term effectiveness of the training course in terms of knowledge retention, implementation of iPSC disease modelling in home institutions and its outcomes on career progression, a follow-up questionnaire in 12 months’ time would be needed and is planned. Previously, Bertalan et al. [16] carried out a longitudinal study utilizing a follow-up questionnaire. This study illustrated the beneficial long-term impact of a training course model with almost 95% of the training course attendees still involved in the field [16]. It would be interesting to investigate if similar effects are observed in our training course model and whether the different groups show varied outcomes. Furthermore, assessing if future grants and collaborations were successful would require a longer-term study of outcomes which is also planned.

## Conclusions

This study highlights the feasibility, effectiveness and high levels of trainee satisfaction delivered by our novel Hands-on and Observer group hybrid teaching method used at the COST training course in Dublin. Trainees of both groups retained a high degree of knowledge of iPSC characteristics and culturing techniques after the training course and the networking opportunities at the training course led to multiple successful scientific missions to host institutions across the ERA. Additionally, we have shown that the use of a hybrid model can have the dual effect of teaching a larger group of researchers while also providing an invaluable resource of video protocols, describing the techniques explored in the training course. We have shown that this new approach to teaching can advance the understanding of novel techniques and encourages the adoption of innovative methods such as iPSC-derived alveolar cells and macrophages to model rare lung diseases. This should lead to the establishment of new research networks and ultimately better treatments for patients.

### Supplementary Information

**Additional file 1. **Training course video: A comprehensive learning video detailing iPSC culturing techniques.

**Additional file 2. **Training booklet: Booklet covering the learning objectives and all the protocols of the practical sessions of the training course.

**Additional file 3. **Questionnaire to determine satisfaction and knowledge of the trainees after the training course.

**Additional file 4. Fig: S1. **Gender distribution of the speakers of the COST Action conference.

## Data Availability

All data generated or analysed during this study are included in this published article [and its additional files].

## Abbreviations

COST: European Cooperation in Science and Technology
ENTeR chILD: European Network for Translational Research in Children’s and Adult ILD
ILD: Interstitial lung diseases
iPSC: Induced pluripotent stem cell
MCQ: Multiple choice question
SRG: Surfactant related genes
TRG: Telomere related genes
WG: Working group
