# Supplementary material for: An evaluation of an open access iPSC training course: “How to model interstitial lung disease using patient-derived iPSCs”
Source: Stem Cell Res Ther. 2023 Dec 20;14:377. doi: 10.1186/s13287-023-03598-9 (PMC10734099; doi:10.1186/s13287-023-03598-9)
Supplement: Supplementary file 2 — Additional file 2. Training booklet: Booklet covering the learning objectives and all the protocols of the practical sessions of the training course. [file 13287_2023_3598_MOESM2_ESM.docx]

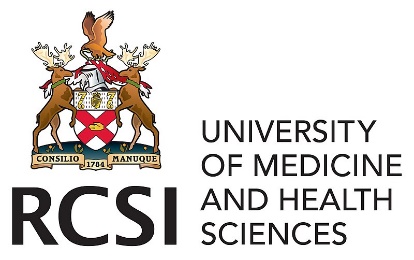

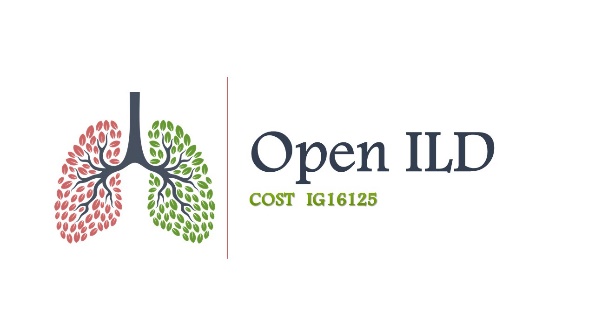

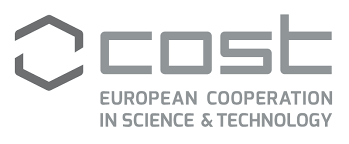


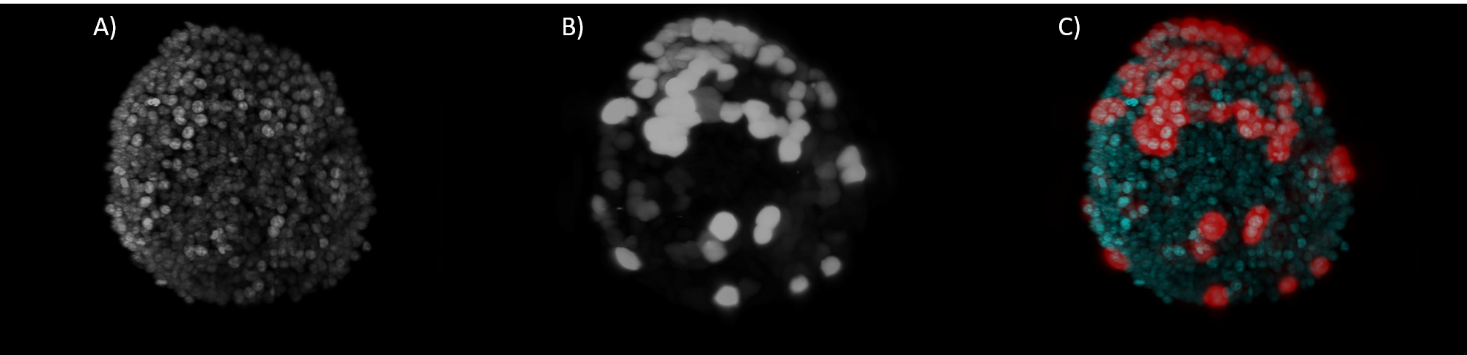

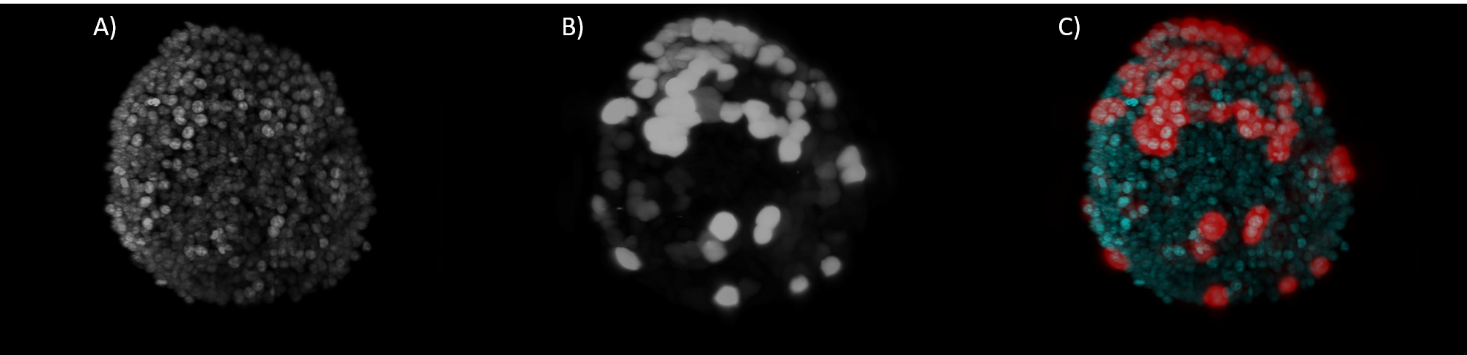

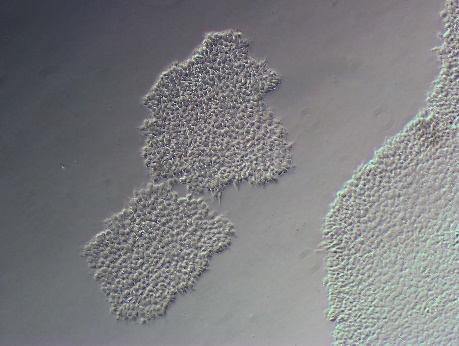

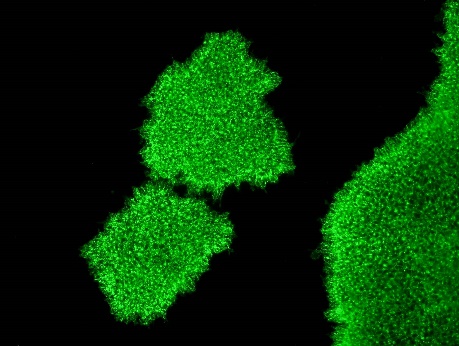

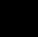


Open-ILD: An Open Access Repository of Pluripotent Stem Cells from Children and Adults with

Interstitial Lung Disease

**COST Action IG16125**

Practical Sessions Manual

RCSI, The Smurfit Building, Beaumont Hospital

25th - 27th May, 2022

Website: www.livinglunglab.ie

| Session | Learning Objective |
| --- | --- |
| A | - To understand the principles and techniques for culturing iPSC based on the introductory lecture. - To understand the practical aspects of (i) how to coat plates with 2D Matrigel for iPSC culture, and (ii) how to passage iPSC on Matrigel using Gentle Cell Dissociation Reagent. |
| B | - To understand the theoretical and practical aspects of thawing frozen stocks of iPSC onto Matrigel. |
| C | - To understand the theoretical and practical aspects of making frozen stocks of iPSC for long term storage. |
| D | - To understand the principles and mechanisms behind generating endoderm and subsequently lung progenitors and distal lung organoids from iPSC using a directed differentiation protocol. - To understand the principles and mechanisms involved in generating macrophages from iPSC. - To understand the practical aspects of preparing/seeding iPSC for differentiation towards endoderm. |
| E | - To recognise the expected microscopic appearance and characteristics of (i) iPSC passaged in Session A; (ii) iPSC thawed in Session B; and (iii) iPSC seeded for endoderm differentiation in Session D |
| F | - To examine by microscope: (i) iPSC-derived macrophages, and (ii) iPSC-derived lung organoids |
|  |  |

# Workflow Diagram

**Filled Boxes:** protocols covered in this training course

**Clear boxes:** additional protocols and end point products of the differentiation

iPSC-derived Macrophages

Alveolar Organoids

Lung Progenitors

Anterior foregut differentiation

Endoderm differentiation

**iPSC culture and maintenance**

Passing iPSCs

Thawing iPSCs

Freezing iPSCs

Prepare Matrigel^TM^ coated Tissue Culture Plates

# Protocols

## **Preparation of Matrigel-coated Tissue Culture Plates for human induced pluripotent stem cell (hiPSC) Culture**

**Notes:** hiPSC cells are cultured on Matrigel^TM^ coated dishes. Matrigel^TM^ is a reconstituted basement membrane extract that gels at room temperature. Matrigel concentration varies depending on Lot No. therefore use the dilution factor provided on the accompanying Cert of Analysis for each different lot received. Thaw stock vial on ice at 4°C overnight and aliquot and store at -80°C for single use only. Aliquots of approx. 270-350µl (depending on lot specific dilution factor) diluted in 25ml of DMEM/F12 is enough for 4 x 6 well plates, (1ml/well). Smaller aliquots are frozen for coating less wells.

<https://certs-ecatalog.corning.com/life-sciences/product-descriptions/356277.pdf>

**Materials and reagents for coating 1x6well plate**

- Corning^TM^ Matrigel^TM^ hESC-qualified Matrix (Corning^TM^ 354277)
- Gibco DMEM/F12 (Gibco^TM^ 31330-038)
- 6-well tissue culture plates (Corning^TM^ 3506, 3513)

1. Thaw appropriate aliquot of Matrigel on ice.
2. Prepare 6ml 4 °C DMEM/F12 in 15 ml Falcon.
3. Add 0.5 ml of cold DMEM/F12 into Matrigel aliquot and carefully transfer back into remaining 5.5ml of cold media, mix gently to avoid creation of bubbles.
4. Coat plates by adding 1 ml of Matrigel-DMEM/F12 solution per well of 6-well tissue culture plate (0.5 ml per well of 12-well tissue culture plate). Gently rock to cover the whole surface of each well.
5. Incubate at 37 °C for 30 to 60 min before use. Take care not to disturb when Matrigel setting to avoid creating an uneven surface.
6. If not using immediately – store plates wrapped in parafilm at 4°C for up to 1 week, NO NEED FOR 1HR INCUBATION IN THIS CASE.

## **Thawing hiPSCs onto Matrigel**

- mTeSR^TM^ Plus (StemCell Cat #100-0276)
- Rock Inhibitor (RI) (Tocris Cat #1254)
- DMEM/F12 (Gibco^TM^ 31330-038)
- Matrigel-coated plate
- 50ml conical tube
- 37°C waterbath

1. Prepare 10 ml mTeSR + 1:1000 RI.
2. Aspirate Matrigel from a coated 6-WP and wash with 1 ml (per well of 6 WP) DMEM/F12, add 2 ml mTeSR + RI per well and return plate to incubator.
3. Bring the frozen hiPSC containing cryovial to the tissue culture room on ice.
4. Quickly thaw the hiPSCs by holding the vial into ~37 °C warm water under gentle movement.
5. Once they start to thaw, wipe the cryovial and spray it with 70 % IMS to place it in the hood.
6. Transfer the hiPSC solution into an empty 50 ml Falcon.
7. Add 5 ml warm mTeSR + RI dropwise to cells while gently swirling the falcon.

- These steps need to be carried out fast, as DMSO is toxic to the cells at room temperature.

1. Centrifuge for 5 min at 200 x g at RT.
2. Aspirate off the medium and resuspend in 1 ml mTeSR + RI (per vial thawed).

- Do NOT pipette! Only gently flick the Falcon to resuspend the cells.

1. Add 0.5 ml of resuspended cells gently dropwise to two wells of 6 WP prepared in step 2 using a Pasteur pipette.
2. Return hiPSCs to the incubator and distribute cells evenly in wells by moving plate in a side to side and back and forth movement.

## **Passaging hiPSCs on Matrigel using Gentle Cell Dissociation Reagent**

In general, hiPSCs should be passed when they are 70-80 % confluent, so when the colonies become large and start touching each other. This density is approximately reached after five to seven days at a 1:5-1:20 split ratio dependent on the cell line. hiPSCs cultured on Matrigel-coated dishes are passaged using non-enzymatic dissociation reagents such as Gentle Cell Dissociation Reagent. Unlike other cell types, iPSCs are passaged as clumps or aggregates.

**Note:** Check cells under microscope to assess and mark areas that have started to differentiate and remove these before passaging cells. Use a marker to define areas to be cleaned up on outside of the culture dish.

- DPBS (Gibco^TM^ 14190094)
- Gentle Cell Dissociation Reagent (StemCell Cat #07174)
- DMEM/F12 (Gibco^TM^ 31330-038)
- mTESR^TM^ Plus (StemCell Cat #100-0276)
- Matrigel-coated plates

1. Preparation of Matrigel-coated plate: aspirate Matrigel-DMEM/F12 solution from a coated well. Wash with 1 ml DMEM/F12 and add 2 ml of fresh mTeSR medium per well of a 6-WP (for 12-WP: wash with 0.5 ml DMEM/F12, add 1 ml of fresh mTesR per well). Return the plate to the incubator.
2. Observe iPSCs under a microscope and mark differentiation.
3. Additional step: remove regions of differentiation with a small pipette tip. Be careful to not disturb nearby hiPSC colonies.
4. Aspirate the medium and wash well with 1 ml of DPBS (if differentiation was removed add one additional washing step with 1 ml of DPBS).
5. Add 1 ml Gentle Cell Dissociation Reagent (GCDR) per well of a 6-WP (0.5 ml per well of a 12-WP) for 5 min at RT. **Use a timer for this step.**
6. Aspirate GCDR and add 1 ml mTESR in a **swirl** to detach cells.

- Important: do not pipette cells up and down as this will break the colonies. If cells aggregates are large gentle pipetting using a 5ml pipette can be performed x1-3 times.

1. Check colony amount and sizes under microscope.
2. Add appropriate amount of cell suspension to well of a prepared Matrigel-coated plate (from Step 1).
3. Return iPSCs to the incubator and distribute them evenly over well.

## **Freezing of hiPSCs Growing on Matrigel**

Before freezing hiPSCs, they should be examined for differentiation. As for passaging, hiPSCs should be around 70-80 % confluent. It is important to freeze the cells quickly after resuspending in freezing solution, as prolonged exposure to DMSO at room temperature will decrease the survival of the cells. Ensure cryovials and freezing media are kept on ice before use. Freeze 1 well (from a 6 well plate) into 1 cryovial.

- DPBS (Gibco^TM^ 14190094)
- Gentle Cell Dissociation Reagent (StemCell Cat #07174)
- mTeSR^TM^ Plus (StemCell Cat #100-0276)
- 2X Freezing media (80% mTeSR^TM^ Plus, 20% DMSO; Sigma 41639-100ML)

1. Observe iPSCs under a microscope and mark differentiation.
2. Additional step: remove regions of differentiation with a small pipette tip. Be careful to not disturb nearby hiPSC colonies.
3. Aspirate the medium and wash well with 1 ml of DPBS (if differentiation was removed add one additional washing step with 1 ml of DPBS.
4. Add 1 ml Gentle Cell Dissociation Reagent (GCDR) per well of a 6-WP (0.5 ml per well of a 12-WP) for 5 min at RT.
5. Aspirate GCDR and add 1 ml mTESR in a **swirl** to detach cells. If cells remain adherent to plate, use a cell scraper to gently detach the cells as aggregates.
6. Transfer floating colonies to 15 ml falcon.
7. Additional step: wash well with 1 ml DMEM/F12 to collect the rest of the cells.
8. Centrifuge for 5 min at 200 x g and aspirate medium.
9. Gently resuspend hiPSCs in mTeSR (0.5 ml mTeSR per vial to freeze) using a Pasteur pipette
10. Add 0.5 ml freezing solution (mTeSR + 20% DMSO; COLD!) dropwise using a Pasteur pipette while swirling the tube to mix.
11. Immediately aliquot 1 ml of cell suspension per cryovial.

- Keep vials on ice and transfer to -80 °C as soon as possible.

1. Place the cryovial in a Styrofoam rack at -80 °C overnight and transfer them to liquid nitrogen the following day. (Careful: Some hiPSC lines are stored at -80 °C for long-term)

##

## **Set Up for Endoderm Differentiation using STEMdiff^TM^ Definitive Endoderm Kit**

Endoderm differentiation is carried out according to the STEMdiff^TM^ Definitive Endoderm Kit. <https://www.stemcell.com/stemdiff-definitive-endoderm-kit.html>. To start differentiation of hiPSCs towards definitive endoderm, the cells have to be seeded as single cells. Rock inhibitor is added to avoid spontaneous differentiation. Different time-points throughout the differentiation can be seen in figure 1.

1. DPBS (Gibco^TM^ 14190094)
2. mTeSR^TM^ Plus (StemCell Cat #100-0276)
3. Rock Inhibitor (RI) (Tocris Cat #1254)
4. Gentle Cell Dissociation Reagent (StemCell Cat #07174)
5. Matrigel-coated 12-WP
6. STEMdiff^TM^ Definitive Endoderm Kit (StemCell Cat #05110)

**Start endoderm differentiation (Day -1)**

1. Preparation of Matrigel-coated plate: aspirate Matrigel-DMEM/F12 solution from a coated 12-WP. Wash with 0.5 ml DMEM/F12 and add 1 ml of fresh mTeSR medium + 1:1000 RI per well. Return the plate to the incubator.
2. Observe iPSCs under a microscope and mark differentiation.
3. Additional step: remove regions of differentiation with a small pipette tip. Be careful to not disturb nearby hiPSC colonies.
4. Aspirate the medium and wash well with 1 ml of DPBS (if differentiation was removed add one additional washing step with 1 ml of DPBS).
5. Add 1 ml Gentle Cell Dissociation Reagent (GCDR) per well of a 6-WP for 10 min at 37 °C.
6. Swirl **in GCDR.**
7. Pipette up and down 3x using P1000 to break up colonies / cell clumps.

- Check under microscope, if still many cell clumps repeating pipetting for 3x.

1. Transfer into 15 ml falcon containing 1 ml DMEM/F12 per well used.
2. Rinse well with 1 ml DMEM/F12 and transfer to the 15 ml falcon.
3. Centrifuge for 5 min at 250 x g.
4. Aspirate media and resuspend in 0.5 ml mTeSR + 1:1000 RI per well of 6 WP.
5. Add 250 µl of cell suspension per well of 12 WP (1 well of 6WP into 2 wells of 12WP) (approx. 1 x 10^6 / well of 12WP) prepared in step 1.

# Resources, Paper and Further Reading

**Definitive endoderm differentiation:** STEMdiff^TM^ Definitive Endoderm Kit: <https://www.stemcell.com/stemdiff-definitive-endoderm-kit.html>

**Anterior foregut differentiation and downstream differentioation:**

McCauley KB et al., 2018 „Derivation of Epithelial-Only Airway Organoids from Human Pluripotent Stem Cells“ DOI: [10.1002/cpsc.51](https://doi.org/10.1002%2Fcpsc.51)

Jacob A et al., 2019 “Derivation of self-renewing lung alveolar epithelial type II cells from human pluripotent stem cells” DOI: [10.1038/s41596-019-0220-0](https://doi.org/10.1038/s41596-019-0220-0)

**iPSC derived macrophages:**

Ackermann M et al., 2022 “Continuous human iPSC-macrophage mass production by suspension culture in stirred tank bioreactors” DOI: [10.1038/s41596-021-00654-7](https://doi.org/10.1038/s41596-021-00654-7)
