# Supplementary material for: An evaluation of an open access iPSC training course: “How to model interstitial lung disease using patient-derived iPSCs”
Source: Stem Cell Res Ther. 2023 Dec 20;14:377. doi: 10.1186/s13287-023-03598-9 (PMC10734099; doi:10.1186/s13287-023-03598-9)
Supplement: Supplementary file 3 — Additional file 3. Questionnaire to determine satisfaction and knowledge of the trainees after the training course. [file 13287_2023_3598_MOESM3_ESM.docx]

**3-Day Training Course Evaluation: Open-ILD: An Open Access Repository of Pluripotent Stem Cells from Children and Adults with Interstitial Lung Disease**

**1. Information about the attendee**

**Your position:**

- Clinician
- Researcher
- Clinician with a research activity in a lab using cell culture
- Clinician with another research activity
- Other

**Your age**

- <40
- >40

**Before attending, did you experimented culture cell?**

- Yes
- No

**Before attending, did you use iPSC?**

- Yes
- No

**2. Survey of Satisfaction**

**Overall satisfaction of the Hands-on training / Observational training (from 0 (not satisfied) to 10 (highly satisfied))**

**Overall satisfaction of the organization of the Hands-on session / Observational session (from 0 (not satisfied) to 10 (highly satisfied))**

**Scoring of satisfaction of the sessions (from 1 (lowest) to 6 (best))**

- Practical Session a – Basics of iPSC Culture and Maintenance 1
- Practical Session b – Thawing iPSCs
- Practical Session c – Freezing iPSC
- Practical Session d – Differentiation of iPSC
- Practical Session e – Evaluation of iPSC Cultures and Differentiation
- Practical Session f – Evaluation of iPSC, Macrophage and Organoids

**Scoring of understanding of the sessions (from 1 (lowest) to 6 (best))**

- Practical Session a – Basics of iPSC Culture and Maintenance 1
- Practical Session b – Thawing iPSCs
- Practical Session c – Freezing iPSC
- Practical Session d – Differentiation of iPSC
- Practical Session e – Evaluation of iPSC Cultures and Differentiation
- Practical Session f – Evaluation of iPSC, Macrophage and Organoids

**3. Self-estimation of knowledge improvement**

**Rate your personal theoretical knowledge improvement after the training (from 0 (no improvement) to 10 (the best improvement I could imagine))**

**Rate your personal practical knowledge improvement after the training (from 0 (no improvement) to 10 (the best improvement I could imagine)**

**4. Knowledge about iPSC culture after the training course**

**How would you describe iPSCs?**

- Pluripotent cells
- Differentiated lung cells
- Differentiated endodermal cells
- Alveolar cells
- I don’t know

**Can iPSC be stored long-term?**

- Yes, at 4 C
- Yes, at -20 C
- Yes, at -80 C or in liquid nitrogen
- No
- I don’t know

**How are iPSCs cultured?**

- On a dry plastic dish (adherent)
- In a liquid medium (suspension culture)
- On a gel (adherent)
- I don’t know

**How are iPSCs cultured?**

- 37 C
- 4 C
- Room temperature
- Room air
- FiO2 30%
- FiO2 50%
- I don’t know

**Which confluence is targeted for iPSC differentiation?**

- 10-20%
- 40-50%
- 70-80%
- 100%
- I don’t know

**Which confluence is targeted for iPSC freezing?**

- 10-20%
- 40-50%
- 70-80%
- 100%
- I don’t know

**What is DMSO used for?**

- Kill differentiated cells to keep only stem cells
- Protection of frozen cells
- Maintenance medium of cultured cells at room air
- I don’t know

**How long does it take to differentiate iPSCs into cells with endodermal characteristics?**

- 1 day
- 3 days
- 7 days
- I don’t know

**What is the purpose of the FACS step after endodermal differentiation of iPSCs?**

- Checking the cell viability
- Checking for endoderm-specific cell surface marker
- Checking for pluripotency cell surface marker
- I don’t know

**What is an organoid?**

- A differentiated organ generated in vitro
- An artificial organ
- Isolated cells generated in vitro into organ-specific cells
- A group of cells generated in vitro and mimicking organ-specific cells
- I don’t know
