# Supplementary figures and images for: An evaluation of an open access iPSC training course: “How to model interstitial lung disease using patient-derived iPSCs”
Source: Stem Cell Res Ther. 2023 Dec 20;14:377. doi: 10.1186/s13287-023-03598-9 (PMC10734099; doi:10.1186/s13287-023-03598-9)

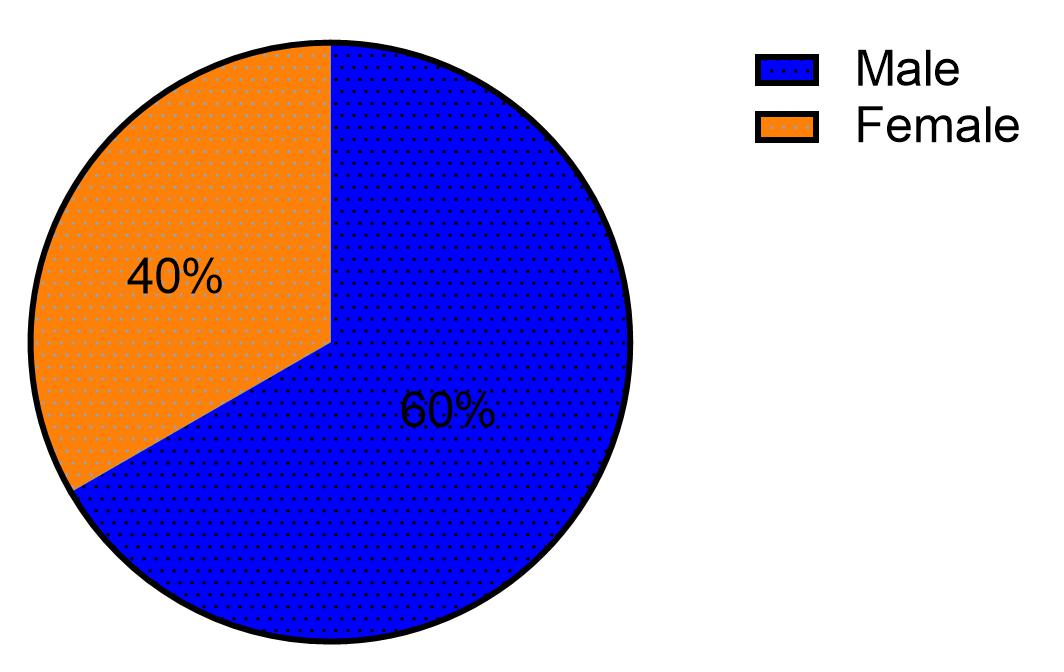

Supplement: Supplementary file 4 — Additional file 4. Fig: S1. Gender distribution of the speakers of the COST Action conference. [file 13287_2023_3598_MOESM4_ESM.png]
